# Radiotherapy for meningiomas

**DOI:** 10.1007/s11060-022-04171-9

**Published:** 2022-10-31

**Authors:** William C. Chen, Haley K. Perlow, Abrar Choudhury, Minh P. Nguyen, Kanish Mirchia, Mark W. Youngblood, Calixto-Hope G. Lucas, Joshua D. Palmer, Stephen T. Magill, David R. Raleigh

**Affiliations:** 1 Department of Radiation Oncology, University of California San Francisco, San Francisco, CA 94143 USA; 2 Department of Neurological Surgery, University of California San Francisco, San Francisco, CA 94143 USA; 3 Department of Radiation Oncology, Ohio State University, Columbus, OH 43210 USA; 4 Department of Pathology, University of California San Francisco, San Francisco, CA 94143 USA; 5 Department of Neurological Surgery, Northwestern University, Chicago, IL 60611 USA; 6 Department of Pathology, Johns Hopkins University, Baltimore, MD 21287 USA

**Keywords:** Meningioma, Radiation, Radiotherapy, Atypical, Anaplastic, Molecular, DOTATATE

## Abstract

Meningiomas are the most common primary central nervous system neoplasm. Despite promising recent progress in elucidating the genomic landscape and underlying biology of these histologically, molecularly, and clinically diverse tumors, the mainstays of meningioma treatment remain maximal safe resection and radiation therapy. The aim of this review of meningioma radiotherapy is to provide a concise summary of the history, current evidence, and future for application of radiotherapy in meningioma treatment.

## A history of radiotherapy for meningioma

The use of X-rays to treat meningioma dates back to the early twentieth century. References to the use of radiotherapy can be found in Cushing and Eisenhardt’s 1938 seminal monograph *Meningiomas *[1], and in Donald Simpson’s influential 1957 surgical series [2]. Early experiences reported a variety of doses and techniques that were generally used sporadically to treat recurrent, refractory or inoperable tumors [2–4]. The rarity of brisk tumor regression led some early investigators to regard meningiomas as insensitive to ionizing radiation. Nevertheless, careful observation and continued interest in radiotherapy over many decades led to multiple institutional series published in the 1970s and 1980s that demonstrated the efficacy of radiotherapy in arresting growth in the majority of meningiomas, and occasionally engendering clear and durable tumor regression [5]. These included influential reports by Wara et al. [6] of the University of California San Francisco (UCSF) who in 1975 observed substantial improvement in recurrence rates with “immediate radiotherapy” following subtotal resection as compared to observation (29% vs 74%). Several similar experiences from investigators at other institutions corroborated Wara’s findings [7–10]. The relatively favorable outcomes reported with the addition of radiotherapy appeared to improve upon the high recurrence rates following subtotal resection alone of meningioma, which ranged from a 44% crude recurrence rate reported by Simpson et al. [2], to 55% and 91% at 10 and 15 years, respectively, reported by Mirimanoff et al. in [11]. In further support of radiotherapy, Smith et al. [12] described the use of X-ray therapy as primary treatment in five patients with optic nerve sheath meningioma in 1981, leading to improvement in visual acuity and/or disease stabilization in all cases. Thus, early reports suggested radiotherapy could be used as a meaningful therapeutic alternative to treat meningiomas in sensitive locations, for which surgical resection carried a high risk of morbidity.

Multiple technological advances in the 1980s and 90s led to the transformation of radiotherapy, driven by improvements in and greater uptake of computed tomography (CT) and magnetic resonance imaging (MRI), major improvements in radiation planning and radiation delivery that coincided with exponential growth in computational capacity, the advent of stereotactic radiosurgery, and rapid expansion of linear accelerator technology. In 1994, Goldsmith et al. [13] reported an update of the earlier series by Wara et al. now comprised of outcomes from 140 patients treated between 1967 and 1990 with a median of 54 Gy after subtotal resection. Goldsmith found substantial improvement in tumor control for patients treated in the more modern era (after 1980, coinciding with use of MR and CT guided radiotherapy at UCSF), with 98% control of benign meningiomas at 5 years after 1980 as compared to 77% prior to 1980. The authors identified a correlation of improved tumor control with minimum tumor doses greater than 52 Gy for benign meningioma. Separately, a report by Glaholm et al. [7] in 1990 found improvement in physician-reported neurological performance status in 38% of inoperable patients treated with primary radiotherapy, underlining the clinical responsiveness of meningioma to radiation. Many of the observations made by these early investigators would come to be reiterated and validated in contemporary reports of radiotherapy for meningioma.

## Efficacy and safety of radiotherapy for meningioma

### WHO grade

Historically, the outcomes and management considerations for meningioma have been stratified based mainly upon a combination of extent of resection and histopathologic grading, which has been standardized within the World Health Organization (WHO) grading system beginning in 1993. This system has undergone several major revisions [14–17] in 2000, 2007, 2016, and 2021 (Table 1). In particular, after institution of more specific criteria for WHO grade 2 and 3 meningiomas in 2000, the distribution of grade 1, 2 and 3 meningiomas underwent a moderate shift, with greater than 90% of meningiomas classified as grade 1 prior to 2000, and approximately 75–80% of meningiomas classified as grade 1 after 2000 [18]. The WHO grading system continues to change alongside the ever-growing knowledge of meningioma biology, tumorigenesis and progression. As such, interpretation of literature for meningioma radiotherapy over different eras must be done with our evolving understanding of WHO grading and meningioma biology in mind.

**Table 1:** Meningioma classification over time by WHO grading criteria

WHO Classification Version	Criteria for WHO grade 2	Criteria for WHO grade 3
1979	No distinction by grade. Meningotheliomatous, fibrous, transitional, psammomatous, angiomatous, hemangioblastic, hemangiopericytotic, and papillary subtypes	No distinction by grade, however anaplastic subtype known to be more aggressive
1993	“Several” of (1) Frequent mitoses (2) Hypercellularity (3) Small cells with high nuclear to cytoplasmic ratio (4) Prominent/pleomorphic nucleoli Hemangiopericytoma distinguished as non-meningothelial entity	Features of frank malignancy far in excess of the abnormalities in atypical meningioma
2000	4–19 mitoses per 10 hpf, OR presence of at least 3 of: (1) Hypercellularity (2) Small cells with high nuclear to cytoplasmic ratio (3) Prominent/pleomorphic nucleoli (4) Sheeting like growth (5) Spontaneous or geographic foci of necrosis	20 or higher mitoses per 10 hpf, OR frank anaplasia with de-differentiation, resemblance to carcinoma, sarcoma, or melanoma
2007	Same as 2000, with addition of chordoid and clear cell subtypes	Same as 2000, with addition of papillary and rhabdoid subtypes
2016	Same as 2007, with the addition of brain invasion as a standalone criterion of WHO grade 2	Same as 2007
2021	Same as 2016. *SMARCE1* mutation associated with clear cell subtype. Methylation classes may be useful for prognosis, but no criteria defined. Roman numerals no longer used	Rhabdoid and papillary subtypes no longer automatic criteria for grade 3 in absence of other criteria. Molecular features associated with aggressive behavior: (1) *BAP1* mutation (2) hotspot *TERT* promoter mutation (automatic criteria for grade 3) (3) homozygous *CDKN2A* deletion (automatic criteria for grade 3)

### WHO grade 1 meningioma

The evidence supporting the safety and efficacy of modern radiotherapy in the treatment of WHO grade 1 meningiomas is substantial. Comprehensive reviews of the literature by Rogers et al. as part of a Response Assessment in Neuro-Oncology (RANO) working group in 2015 [19], and by Maclean et al. in 2014 [20], reported on sequential series published between 1983 and 2012 containing over 2000 patients treated with radiotherapy, largely for benign meningiomas, and most commonly to a dose of 50–55 Gy. Rates of tumor control among studies published after the year 2000 ranged between 76 and 100%, with most reporting rates between 90 and 95%. Furthermore, the rate of clinical improvement was noted between 20 and 81% after radiotherapy, with most studies reporting rates between 30 and 40%. Multiple studies have reported meningioma regression of approximately 30–35% in size [21–23], with most reduction occurring within the first 2–3 years after radiotherapy. The proportion of meningiomas reducing after radiotherapy has been reported to be between 1 and 46%, with most studies reporting rates between 20 and 30%. Thus, the observation of growth arrest following radiotherapy in the great majority of benign meningiomas—and of cases with durable clinical responses and meningioma regression—which were initially made decades ago by early investigators have been well borne out in the modern literature.

Further compelling evidence of the efficacy and safety of radiotherapy can be found in more recent series reporting outcomes of optic nerve sheath and cavernous sinus meningiomas, for which radiotherapy often has a central role in management. A systematic review and meta-analysis by de Melo et al. [24] in 2021 accumulated 736 cases from 39 sequential series of optic nerve sheath meningioma between 1981 and 2019 and found a pooled local control rate of 97.4%, rate of improved visual acuity of 45%, and stable or improved acuity of 85%. The probability of vision improvement seemed to increase in recent studies using modern technologies such as 3-dimensional conformal radiotherapy (3D-CRT) or intensity modulated radiotherapy (IMRT). Moreover, rates of sequelae such as optic neuritis, retinopathy, iritis and dry eye, appear to have substantially reduced to 0–6.6% with the use of IMRT or SRS.

Leroy et al. [25] performed a systematic review and meta-analysis of radiotherapy for cavernous sinus meningiomas in 2018, including 420 patients treated with fractionated radiotherapy, and found a 5-year and 10-year progression freedom of 97.4% and 95.5%, respectively. Oculomotor and trigeminal nerve function improved in 53% and 54.5% of patients, respectively, after fractionated radiotherapy to a median of 51.2 Gy. Adverse radiation related effects were rare, occurring in less than 5% of cases.

Despite these encouraging observations, prospective data regarding WHO grade 1 meningiomas is scarce. RTOG 0539, a non-randomized Phase II study, stratified meningioma patients by clinical risk groups: clinically low-risk patients with primary WHO grade 1 meningiomas underwent observation [26]. Within this population, the 5-year progression free survival of patients with subtotally resected WHO grade 1 meningiomas was 72.7%. Similarly, in the multicenter propensity-matched IMPASSE study [27], imaging defined meningiomas that were presumed to be benign and underwent observation experienced tumor control of 64.2% at a mean of 43.5 months of follow up. Thus, a significant subset of patients with WHO grade 1 meningiomas remain at risk of recurrence with or without resection, but the ideal timing of and indications for radiotherapy, and the optimal factors for selection of patients for escalated management, remain subject to debate. Recent and future developments in molecular characterization and imaging of meningioma [28] promise to bridge these gaps in knowledge and allow for more individualized selection of patients for radiotherapy or other forms of intensified management.

### WHO grade 2 meningioma

Atypical WHO grade 2 meningiomas are less common, comprising 15–25% of these lesions. Nevertheless, the evidence supporting the safety and efficacy of radiotherapy for the treatment of these tumors after gross total or subtotal resection has accumulated over the past several decades and is now substantial, including outcomes from two prospective non-randomized trials: RTOG 0539 [29] and EORTC 22042-26042 [30].

RTOG 0539 opened for enrollment in 2009 and ultimately included 36 evaluable patients with primary WHO grade 2 meningiomas status post gross total resection and 16 patients with recurrent WHO grade 1 meningiomas with any resection, all of whom received 54 Gy in 30 fractions of radiotherapy as part of the intermediate-risk clinical arm. Central review of pathology, post-operative imaging for extent of resection, and post-hoc central review of radiotherapy target volumes and organs at risk were mandated. Although not mandated, 84.6% of patients received IMRT, reflecting modern practice. RTOG 0539 met its primary endpoints, reporting 3-year progression free survival of 93.8% and local recurrence rate of 4.1% for intermediate risk meningiomas, both of which are notable in the context of 5-year progression free survival of 72.7% for patients with WHO grade 1 meningiomas who underwent postoperative observation in the low-risk clinical arm, as well as in relation to the historical control rate of 3-year progression free survival of 70%. Radiotherapy was well tolerated for intermediate-risk patients, with no grade 3 or higher toxicities that were attributable to radiation.

**Fig. 1:**
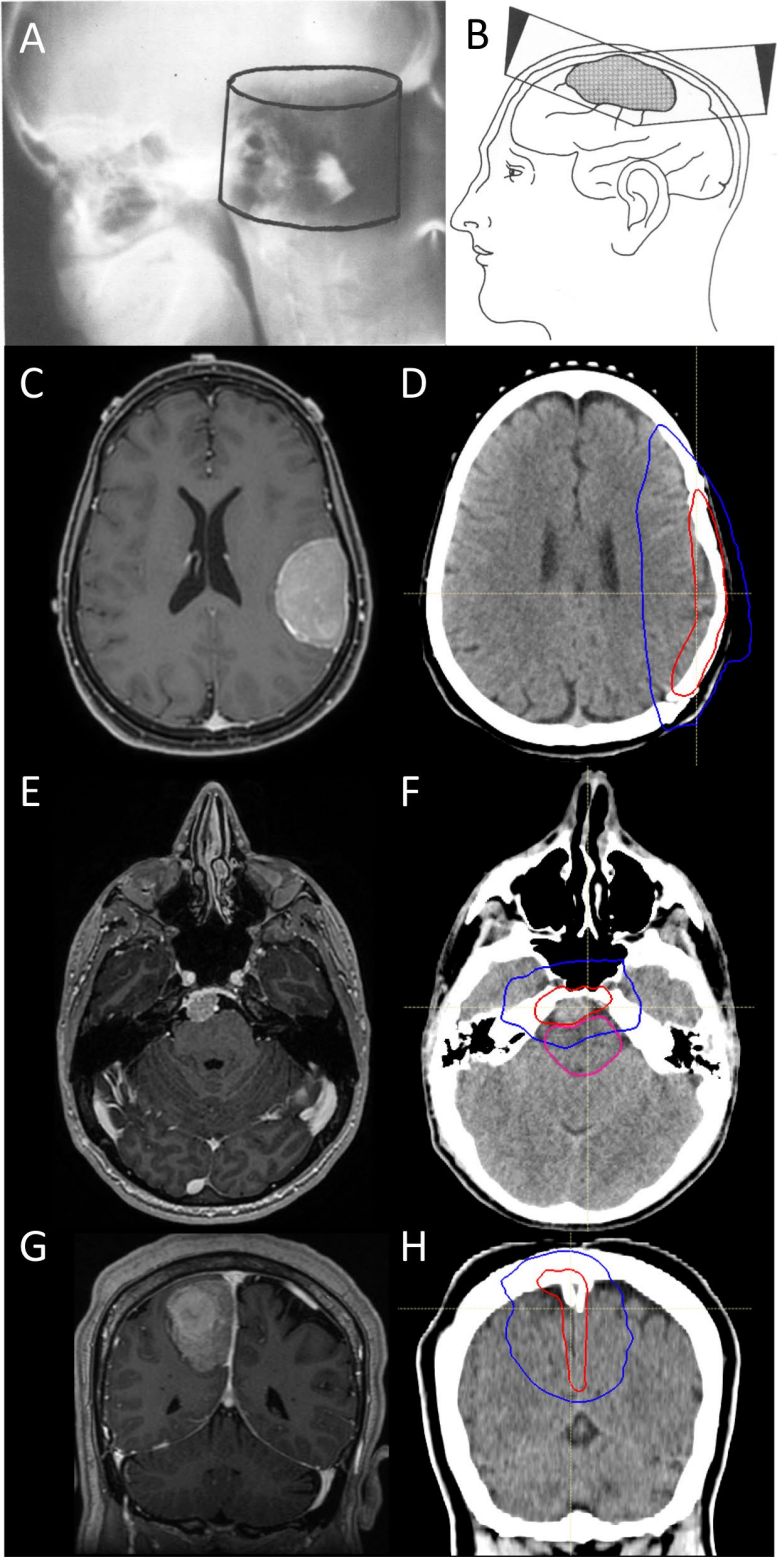
Evolution of meningioma radiotherapy. **A**–**B** Figures reproduced from Friedman et al. 1977 [5]. **A** shows an early example of meningioma radiotherapy. Shown is a 2D film described as a verification “post film” showing a cylindrical irradiated volume targeting a large posterior fossa meningioma, treated sometime before 1963. This volume received a maximum dose of 8000 rad (80 Gy) in 42 days with 2MV photons, a significantly higher dose than reported by most investigators, past or present. This patient was reportedly alive and well 4 years post-radiation. **B** shows a diagram from the same historical publication showing a mock-up of a 2D technique using tangential fields with physical wedges for treatment of a parasagittal meningioma. **C**–**D** shows an axial post-contrast T1 MRI and IMRT plan delivering 59.4 Gy in 33 fractions, respectively, for a large 4.8 cm left frontoparietal meningioma. Gross total resection was achieved, and pathology revealed 5 mitoses per 10hpf, foci of necrosis, hypercellularity and small cell change. No brain invasion was identified, and Ki67 labeling index was 5%. Immunohistochemistry staining showed retained H3K27me and BAP1. An institutional targeted DNA sequencing panel revealed monosomy 22q and a pathogenic *NF2* mutation, along with loss of 1p, 10p and 14q, consistent with high molecular risk. In D), the red line denotes the 59.4 Gy prescription isodose, and blue denotes the 50% isodose line. The target included a customized anisotropic margin of at-risk dura of up to 15 mm, and no explicit clinical target volume expansion into brain, given the absence of brain invasion. **E**–**F** shows an axial post-contrast T1 MRI of an imaging defined, presumed benign meningioma abutting the brainstem, which was treated with 54 Gy in 30 fractions (red isodose line). Treatment was well tolerated. **G**–**H** shows coronal post-contrast T1 MRI and IMRT plan delivering 59.4 Gy in 33 fractions, respectively, for a large, heterogenous and multilobulated meningioma of the posterior falx, which underwent a gross-total resection. Pathology revealed 11 mitoses per 10 high powered fields, elevated Ki67 labeling index of 7%, foci of necrosis, small cell change, consistent with WHO grade 2. Immunohistochemistry showed weak progesterone receptor staining in ~ 25% of cells and retained H3K27me3. An institutional targeted DNA sequencing panel showed no pathogenic SSVs, but chromosomes 22q (*NF2*), 1p, and 19q were lost, consistent with high molecular risk. The target included a customized anisotropic margin of up to 15 mm of at-risk falx and dura, including the sagittal dural sinus abutted by tumor. Treatment was well tolerated, and the patient remains disease free 1.5 years post-therapy

In similar fashion, EORTC 22042-26042 enrolled 56 patients with newly diagnosed WHO grade 2 meningioma status post gross total resection who received 60 Gy in 30 fractions. Again, the primary endpoint was met: 3-year progression free survival was 88.7%, statistically higher than that of the historical control estimate of 70%. Notably, the rate of 3D-CRT use was higher in this study than RTOG 0539 (46.4%), and post-hoc central review of targets/organs at risk was not mandated. Perhaps unsurprisingly, the rate of late grade 3–4 toxicities that could be attributed to radiotherapy in EORTC 22042-26042 was comparably higher than reported in RTOG 0539. Indeed, 5 of 56 patients (8.9%) with WHO grade 2 meningioma who were treated on EORTC 22042-26042 developed late grade 3–4 toxicities, including one patient with optic neuritis and retinopathy, three patients with seizures, and one patient experiencing cerebral ischemia within the radiation field. Dose volume measures in relation to toxicity were not reported. Notably, the study protocol delineated a clinical target volume (CTV-1) to receive 60 Gy which was to encompass suspected areas of subclinical disease, suspicious dural enhancement or thickening, “peritumoral edema”, and hyperostotic changes, altogether with a 10 mm isotropic expansion. Inclusion of peritumoral edema is not a common practice for modern meningioma radiotherapy, and almost universally results in larger volumes of uninvolved brain parenchyma receiving high radiation doses. The permitted use of isotropic CTV margin expansions in both RTOG 0539 and EORTC 22042-26042 also likely exposed more brain parenchyma to high dose radiation than necessary, particularly in the modern era when static and dynamic forms of IMRT are able to shape radiation with high conformality and spare adjacent organs at risk (Fig. 1). Furthermore, controversy exists regarding the significance of brain invasion in otherwise benign appearing meningioma [31], and true recurrence within brain parenchyma appears to be rare except among malignant or multiply recurrent, refractory tumors [32]. Nevertheless, RTOG 0539 and EORTC 22042-26042 comprise the highest levels of evidence available in support of the use of radiotherapy for WHO grade 2 meningiomas in the modern era, incorporating recent WHO grading criteria, routine MRI imaging for surgical and radiotherapy planning and post-operative evaluation, and more modern techniques of radiotherapy delivery. These two trials form the basis of two ongoing Phase 3 randomized trials of radiotherapy versus observation following gross total resection of WHO grade 2 meningioma: NRG BN-003 and ROAM/EORTC-1308.

The prospective evidence above is further supplemented by numerous observational studies at the institutional level, summarized in two recent meta-analyses and systematic reviews. Song et al. [33] accumulated 24 studies reporting on 3078 patients with WHO grade 2 meningioma, finding that post-operative radiotherapy improved progression free survival regardless of resection extent with a pooled hazard ratio of 0.41 (95% confidence interval 0.30–0.55) after subtotal resection, and 0.73 (95% confidence interval 0.52–0.92) after gross total resection. Similarly, Chun et al. [34] identified 25 studies reporting outcomes of WHO grade 2 meningiomas following gross total resection, comprised of 1232 patients not receiving postoperative radiotherapy and 384 patients receiving postoperative radiation. Again, postoperative radiotherapy improved local recurrence and progression free survival, with pooled hazard ratios of 0.50 (95% confidence interval 0.36–0.68) and 0.66 (95% confidence interval 0.51–0.84), respectively.

Not all institutional or multi-institutional series have reported a benefit to radiotherapy for WHO grade 2 meningioma [35], perhaps owing to the vicissitudes of observational and retrospective research, which could take the form of heterogeneity in selection criteria and practices leading to bias, differing time periods, small sample sizes and short follow up durations, changes or differences in radiation target delineation, dose or delivery technique, and shifts in WHO grading over time. Furthermore, few studies distinguish between local and marginal or out of field failures, nor describe the extent of surrounding dura at risk targeted with adjuvant radiotherapy. With these limitations that are relevant to the majority of meningioma radiotherapy studies in mind, several contemporary investigations are worth highlighting.

Bray et al. in [36] reported outcomes of 162 patients with WHO grade 2 meningiomas that were resected between 1996 and 2018 at Emory, 108 of whom received adjuvant radiotherapy resulting in substantially greater recurrence freedom of approximately 90% at 5 years compared to approximately 50–60% after surgery alone. This study is notable due to the high rate of radiotherapy use. Moreover, 89% of patients received greater than 59 Gy, with the majority of patients receiving 59.4 Gy, reflecting the most standard modern practices. Investigators at Emory have previously reported on institutional radiation planning parameters used in the treatment of meningiomas [37], reflecting a systematic approach in treating these tumors. These factors may all contribute to the high rate of tumor control reported.

In 2019, Lee et al. [38] from Yonsei University reported outcomes on 98 patients with WHO grade 2 or 3 meningiomas resected between 2000 and 2013, 58 of whom received post-operative radiotherapy. Notably, the authors carefully detailed target delineation and radiation parameters, including targeting a 1.5–2 cm anisotropic margin of dura at risk, and simultaneous integrated boost of gross tumor residual to 66 Gy. Including WHO grade 3 meningiomas, local control at 5 years was 86.7% with radiotherapy versus 59.3% without, and progression free survival at 5 years was 73.5% versus 54.9%, respectively. Another group of investigators [39] more recently expanded on this experience with a multi-institutional study across 4 centers in Korea which included 518 patients with WHO grade 2 meningiomas resected between 1998 and 2018, 158 of whom received adjuvant radiotherapy, again finding improved progression freedom with radiation (80.8% at 5 years compared to 57.7% after surgery alone). Radiotherapy improved outcomes following gross total resection, as well as across all clinical risk strata devised by the authors.

Chen et al. [40] in 2019 reported remarkably similar results among 182 patients with primary WHO grade 2 meningiomas resected at UCSF between 1993 and 2014, of whom 42 received adjuvant radiotherapy to a median of 59.4 Gy. In this study, 5-year overall freedom from recurrence was 82% with radiotherapy versus 65% without. Thus, with adequate dose, technique, and modern radiotherapy delivery, tumor control at 5-years of 80–90% or higher is achievable among patients with WHO grade 2 meningiomas, an apparent substantial improvement on the known high background rate of recurrence of these tumors after surgery alone. In recognition of the substantial evidence for its efficacy, both the European Association of Neuro-Oncology [41] (EANO) and National Comprehensive Cancer Network [42] (NCCN) guidelines recommend radiotherapy following subtotal resection of WHO grade 2 meningiomas, and consideration of radiotherapy following gross total resection. Future developments in molecular risk stratification of meningiomas may further improve the ability to appropriately select patients for adjuvant therapy, and also promises to allow for identification of patients with favorable tumor biology for whom recurrence may be unlikely. Moreover, future work is needed in understanding the radiobiology of these tumors and the role, if any, of selective dose escalation of areas of macroscopic disease beyond 59.4–60 Gy, along with other strategies to improve outcomes for this patient population who are at substantial risk of tumor related morbidity and mortality.

### WHO grade 3 meningioma

WHO grade 3 meningiomas, also known as anaplastic or malignant meningiomas, are rare, aggressive tumors that are prone to multiple recurrences, invasion, and even extra-cranial metastasis in approximately 8.9% of cases [43, 44]. Combined therapy with maximal safe resection and adjuvant radiotherapy is uniformly recommended by available guidelines [41, 42]. Given the propensity of these tumors to invade adjacent dura, bone, and in some cases brain, adequate margins should be incorporated into radiation design and balanced with the risks of normal tissue toxicity on a case by case basis. Outcomes are poor even with maximal therapy, with 5-year progression free survival of 58.2% reported among newly diagnosed WHO grade 3 meningioma patients in RTOG 0539 following surgery and 60 Gy of radiotherapy [45], and 3- to 5-year progression freedom rates of 8.7–61% [19, 46] reported in numerous small institutional series with variable use of radiotherapy. New treatments informed by meningioma biology and insights into predictive biomarkers or mechanisms to overcome treatment resistance are urgently needed for this challenging disease.

The role of dose escalation beyond 60 Gy for high grade meningiomas has been examined by a handful of investigators who have recognized the predominance of in-field failures for these aggressive tumors [32, 38, 47, 48]. Multiple strategies have been employed. As intimated above, Lee et al. [38] utilized simultaneous integrated boost and IMRT to dose escalate gross residual tumor to 66 Gy, and reported local control in 3 of 4 primary WHO grade 3 meningiomas, without any serious toxicity. Chan et al. [49] utilized a mixed photon and proton therapy approach to dose escalate gross residual meningiomas to 68.4–72 Gy, achieving local control in 4 of 4 WHO grade 2 residual tumors and 1 of 2 WHO grade 3 residual tumors, again without any adverse safety signals. Pontoriero et al. [50] in 2022 reported use of a stereotactic radiosurgery boost in addition to IMRT to achieve an equivalent dose in 2 Gy fractions of 72.5 Gy to areas of gross residual WHO grade 2 meningiomas, reporting no serious toxicities among 16 patients. All 7 patients with primary WHO grade 2 meningioma and gross residual tumors after surgery achieved local control, but 4 of 9 patients with recurrent WHO grade 2 tumors developed in-field failures, underlining the fact that recurrent meningiomas are biologically more aggressive [51] and may frequently encode genetic and epigenetic alterations leading to treatment resistance [52]. Finally, historical series have also noted trends towards improved local control of high grade meningiomas with doses greater than 60 Gy in Boskos et al. [53] and Hug et al. [54], and greater than 53 Gy in Goldsmith et al. [13]. Altogether, the literature examining dose response and meningioma radiobiology remains scarce, and further research is urgently needed, especially for high grade meningiomas.

## Future directions

### Understanding meningioma biology to guide risk stratification

Remarkable progress has been made in the past decade in understanding the genomic and epigenetic landscape of meningiomas. A full review of meningioma biology is beyond the scope of this review, but several key studies and insights are worth highlighting in the context of patient risk stratification. Broadly, informative features can be categorized into recurrent short somatic variants (SSV), chromosomal and genomic instability resulting in somatic copy number variants (CNV), epigenetic subgroups derived from DNA methylation profiling or chromatin immunoprecipitation sequencing [55], and transcriptomic subgroups or signatures (Table 2).

**Table 2:** Selected molecular classification features of meningiomas

Somatic variants	Favorable: *SMO, SUFU, POLR2A, TRAF7, KLF4, PIK3CA* Intermediate: *NF2*, *SMARCE1* Unfavorable: *BAP1*, *TERT* promoter, *CDKN2A/B* deletion, *DMD*, *ARID1A*
Methylation	Sahm et al.: 6 subgroups, ranging from benign to malignant Nassiri et al.: Benign (favorable), immunogenic, hypermetabolic, proliferative (unfavorable) Choudhury et al.: Merlin-intact (favorable), Immune-enriched, Hypermitotic (unfavorable)
Integrated systems	Driver et al.: + 1 points for CDKN2A/B loss (hetero or homozgous), 4–19 mitoses per 10 hpf, and for each of 1p, 3p, 4p, 6p, 6q, 10p, 14q, 18p, 18q, 19p loss + 2 points for 20 or greater mitoses per 10 hpf Low risk = 0–1 points Intermediate risk = 2–3 points High risk = 4 + points

Exome sequencing of meningiomas and matching blood samples [56–58] has identified a plethora of recurrent SSVs ranging from common to rare in the following genes: *NF2, KLF4, TRAF7, PIK3CA, AKT, SMO, SUFU, POLR2A, SMARCB1, SMARCE1, TERT *[59] (promoter), *BAP1* [60], *DMD* [61]*, ARID1A*, and *CDKN2A/B* [62] (heterozygous or homozygous deletion). Broadly, *NF2* SSVs with or without chromosome 22q deletion affecting the *NF2* locus are by far the most common genomic alteration, with biallelic *NF2* inactivation affecting 40–60% of meningiomas. Targeted and exome sequencing has identified SSVs involving the remaining genes in up to 20–40% of meningiomas [56], however this percentage is reduced among cohorts that are enriched for higher grade samples. Consequently, approximately 20–40% of meningiomas lack a previously-reported “driver” event based upon SSV analysis alone. Moreover, SSVs appear to provide modest prognostic power beyond WHO grade, and the most common non-*NF2* SSVs, including *TRAF7, KFL4, AKT, SMO, SUFU, POLR2A, PIK3CA*, are highly correlated with benign appearing histology (WHO grade 1), favorable outcomes, limited genomic instability, and midline skull base location [65]. Conversely, some rare SSVs, such as alterations in the *TERT* promoter, *BAP1, ARID1A, DMD, SMARCE1*, or *CDKN2A/B* deletion, are highly correlated with elevated WHO grade, along with predictably more aggressive imaging and clinical features and less favorable outcomes. Though not yet rigorously studied, none of the SSVs identified have provided markers for meningioma radiotherapy response or resistance, and mechanisms underlying their presumed importance in tumorigenesis and progression remain poorly understood.

Genomic and chromosomal instability has long been correlated with high grade meningioma [66]. Numerous chromosomal regions appear to be recurrently affected by CNVs in meningiomas, most commonly loss of chromosome arm 22q, which contains the *NF2* locus [64, 67]. Loss of 1p (prevalence 9–36%) or 14q (prevalence 15–19%) have also been associated with more aggressive features and poor outcomes. The co-occurrence of 22q loss with 1p loss appears to be a consistent poor prognostic marker, while presence of 22q loss without 1p loss appears associated with intermediate outcomes, and possibly an immune-enriched phenotype as delineated by DNA methylation grouping [52]. Numerous other CNVs have been correlated with aggressive biology, though assessment of their independent significance is limited by low frequency and co-occurrence with 22q and/or 1p loss. A prognostic system devised by Driver et al. [64] incorporating multiple recurrence CNVs along with mitotic index and *CDKN2A/B* loss in a supervised fashion appears to provide additional prognostic power beyond WHO grade alone.

Finally, DNA methylation profiling has emerged as a powerful tool in the classification of central nervous system neoplasms, and its application to meningioma has revealed what appear to be prognostically and biologically meaningful subgroups of meningiomas. Likely due to heterogeneity in study populations and technical differences in data analysis, the optimal number of methylation clusters has ranged from two and six in various studies, including large cohorts reported by Olar et al. [68], Sahm et al. [69], Nassiri et al. [63], and Choudhury et al. [52]. An example technical variation is consideration of CNV status in methylation classification, as these events may confound DNA methylation processing and clustering [52]. Nevertheless, Nassiri et al. and Choudhury et al. both reported at least 3 groups of meningiomas with similar biological characteristics. Both studies identified so-called “benign” or “Merlin-intact” meningiomas that have at least one functional copy of *NF2,* limited genomic instability, and favorable outcomes. Initial studies suggest these lesions may be sensitive to cytotoxic therapy including ionizing radiotherapy. A second group of “Immune-enriched” meningiomas was distinguished by inactivation of *NF2* without concurrent 1p loss, intermediate outcomes, and marked immune infiltrate. The remaining meningiomas were distinguished by enrichment of pathways associated with lipid or nucleotide metabolism, termed “Hypermetabolic”, or “Proliferative” and “Hypermitotic” meningiomas that were enriched in cell cycle and proliferative gene programs such as the *FOXM1* program, and associated with near uniform loss/inactivation of *NF2,* genomic instability with accumulation of high risk CNVs such as 1p loss. Consequently, they exhibit the most aggressive clinical course among described subgroups.

The advances summarized above, along with independent transcriptomic approaches [70–72] that validate DNA methylation-based groups, appear to provide additional prognostic value beyond WHO grade and clearly inform future research into therapeutic vulnerabilities and strategies for meningioma. Nevertheless, predictive biomarkers to guide application of adjuvant radiotherapy remain poorly studied and represent a significant unmet need in the fields of meningioma research and treatment.

### Molecular imaging

The vast majority of meningiomas of all grades express somatostatin receptor 2A (SSTR2A) [73], which presents a convenient target for molecular imaging and theranostics using octreotide peptide analogue radioligands such as 68 Ga- DOTATATE/DOTATOC positron emitting tomography (PET). These innovative imaging approaches appear to enhance sensitivity compared to contrast-enhanced MRI alone in detecting meningioma involvement of dura, dural sinus, and/or bone. One study correlating 68 Ga DOTATATE PET with intraoperative sampling showed greater sensitivity as compared to contrast enhanced MRI (92.3% vs 79.5% for primary and 88.1% vs 76.7% for recurrent tumors). Remarkably, in another study by Bashir et al. [74], residual [68] Ga-DOTATOC PET uptake was noted after gross total resection of 23 of 37 (62%) meningiomas, as determined by standard of care MRI. Of these 23, nine (39%) were confirmed either by subsequent recurrence, or by sampling during re-operation. These rates of occult DOTATATE positive foci following apparent gross total resection mirror histopathologic studies examining meningioma-adjacent dura [75–79], which appear to consistently identify meningioma cells invading between 1.5 and 3 cm away from the tumor edge in between 50–75% of meningiomas. Indeed, these imaging and histopathologic findings corroborate the long-understood relationship between extent of dural resection, as originally classified by the Simpson grade, and recurrence risk. Though not rigorously studied, it is likely that tumor biology influences the presence and extent of dural invasion, as well as the likelihood of these microscopic deposits to proliferate and result in progression.

Some have begun to investigate the use of SSTR2A based molecular imaging to guide radiation planning and target delineation [80, 81], demonstrating the feasibility of reducing target size using customized margins as determined by DOTATATE PET. In light of the propensity of meningioma to infiltrate nearby dura and bone, care should be taken against omitting microscopic residual disease which is below the DOTATATE PET detection limit, which is as yet not well defined. Nevertheless, use of SSTR2A-directed molecular imaging promises to improve the detection of occult residual tumor after resection, particularly for tumors with osseous involvement, in the skull base, or in parasagittal or parafalcine locations, providing further information with which to determine optimal adjuvant management. A summary of the available evidence for PET imaging in meningiomas by the RANO/PET Working Group in 2017 [81] noted 68 Ga DOTATATOC PET or amino acid PET to be particularly useful in radiation planning for improving gross target volume (GTV) delineation and dose sparing of organs at risk, as well as for distinguishing between tumor and post-treatment changes, giving these recommendations an evidence level of 2. Future studies and prospective evaluation of the role of 68 Ga DOTATATOC/DOTATATE PET in radiotherapy planning and meningioma imaging and follow up will likely further clarify the effectiveness of this tool.

## Conclusions

Radiotherapy remains a core component of comprehensive management of meningioma, and substantial evidence supports its role as an effective and safe treatment in both definitive and post-operative settings. Growing understanding of meningioma biology and integration of molecular characterization and SSTR2A-directed imaging promise to improve personalization of risk stratification and management of meningioma patients.
